# The buffer capacity of airway epithelial secretions

**DOI:** 10.3389/fphys.2014.00188

**Published:** 2014-06-03

**Authors:** Dusik Kim, Jie Liao, John W. Hanrahan

**Affiliations:** ^1^Department of Physiology, McGill UniversityMontréal, QC, Canada; ^2^McGill University Health Centre Research InstituteMontréal, QC, Canada

**Keywords:** CFTR, bicarbonate secretion, cystic fibrosis, airway submucosal glands

## Abstract

The pH of airway epithelial secretions influences bacterial killing and mucus properties and is reduced by acidic pollutants, gastric reflux, and respiratory diseases such as cystic fibrosis (CF). The effect of acute acid loads depends on buffer capacity, however the buffering of airway secretions has not been well characterized. In this work we develop a method for titrating micro-scale (30 μl) volumes and use it to study fluid secreted by the human airway epithelial cell line Calu-3, a widely used model for submucosal gland serous cells. Microtitration curves revealed that HCO^−^_3_ is the major buffer. Peak buffer capacity (β) increased from 17 to 28 mM/pH during forskolin stimulation, and was reduced by >50% in fluid secreted by cystic fibrosis transmembrane conductance regulator (CFTR)-deficient Calu-3 monolayers, confirming an important role of CFTR in HCO^−^_3_ secretion. Back-titration with NaOH revealed non-volatile buffer capacity due to proteins synthesized and released by the epithelial cells. Lysozyme and mucin concentrations were too low to buffer Calu-3 fluid significantly, however model titrations of porcine gastric mucins at concentrations near the sol-gel transition suggest that mucins may contribute to the buffer capacity of ASL *in vivo*. We conclude that CFTR-dependent HCO^−^_3_ secretion and epithelially-derived proteins are the predominant buffers in Calu-3 secretions.

## Introduction

Healthy airway epithelium is covered by a microscopic layer of airway surface liquid (ASL) that is overlaid with patches of mucus. The ASL warms and humidifies inspired air and enables cilia on the epithelial cells to beat and clear mucus and particulates from the lungs. Acidification of the ASL occurs in cystic fibrosis (CF) and other inflammatory diseases including asthma (Hunt et al., 2000), chronic obstructive pulmonary disease (COPD; Kostikas et al., 2002), and acute respiratory distress syndrome (Gessner et al., 2003). The airways may also receive acid loads during gastroesophageal reflux and from acidic pollutants (e.g., fog, sulfur and nitrogen dioxide, sulfuric and nitric acid, chlorine gas and hypochlorous acid), which can lead to cell swelling and intercellular edema when the luminal pH falls below ~6.5 (Holma, 1985). Acidification also increases mucus viscosity (Holma, 1989), nitric oxide levels (Gaston et al., 2006) and sodium channel activity (Garland et al., 2013), reduces ciliary beat frequency (Clary-Meinesz et al., 1998) and bacterial killing (Pezzulo et al., 2012), and may trigger bronchoconstriction and cough (Kollarik et al., 2007). These derangements emphasize the importance of pH in this microscopic compartment.

The impact of an acute acid load on ASL pH depends on its buffer capacity. While there have been extensive studies of other exocrine secretions such as saliva, the pH of which is a determinant of tooth decay (Izutsu, 1981; Bardow et al., 2000; Lamanda et al., 2007), less is known regarding the buffer properties of ASL. The buffer capacity of expectorated sputum from cigarette smokers varies linearly with protein content and is ~6 mM/pH with a typical protein concentration of 10 mg/ml (Holma and Hegg, 1989). However sputum is thought to contain proteins, DNA and other macromolecules at higher concentrations than in gland secretions and ASL bathing the epithelial surface.

Most ASL originates as submucosal gland secretions (Trout et al., 2001). The pH of fluid produced by normal and CF glands is 7.2 and 6.6, respectively (Song et al., 2006). Secretions have also been studied using Calu-3, a human adenocarcinoma cell line widely used as a model for submucosal gland serous cells. Like native glands they secrete Cl^−^, HCO^−^_3_, and macromolecules, however there are conflicting data concerning the dependence of HCO^−^_3_ secretion on CFTR. It has been reported that the pH and bicarbonate concentration of Calu-3 secretions are not affected by shRNA knock down of CFTR expression or the CFTR inhibitor GlyH-101 (Garnett et al., 2011), leading to the conclusion that most HCO^−^_3_ secretion is mediated by the anion exchanger pendrin (SLC26A4). Another study of Calu-3 under pH stat conditions found a strong dependence of HCO^−^_3_ secretion on CFTR expression (Shan et al., 2011, 2012), consistent with previous evidence for CFTR-dependent HCO^−^_3_ secretion by Calu-3 cells (Devor et al., 1999; Tamada et al., 2001), primary bronchial cell cultures (Smith and Welsh, 1992) and isolated submucosal glands (Song et al., 2006). However pH-stat conditions require a large basolateral-to-apical HCO^−^_3_ gradient which might exaggerate the contribution of CFTR.

The purpose of this study was to develop a microtitration method suitable for small volumes and use it to study the buffer properties of Calu-3 secretions and the CFTR dependence of HCO^−^_3_ secretion under non-pH stat conditions i.e., without imposing a transepithelial HCO^−^_3_ gradient. We also determined titration curves for individual constituents in the fluid at physiological concentrations to assess their possible contributions to buffer capacity.

## Methods and materials

### Solutions

The following solutions were prepared in distilled water immediately before use: 10, 20, 30, 40, and 50 mM NaHCO_3_; 1 and 10 mM KH_2_PO_4_; 0.5, 1, 500, and 1000 μg/ml porcine gastric mucins; 10 and 50 mg/ml bovine serum albumin. Reagents were from Sigma-Aldrich (St. Louis, MO) and were of the highest grade available.

### Cell culture and collection of secretions

The parental Calu-3 cell line (ATCC, HTB-55) and a CFTR knockdown Calu-3 cell line (Palmer et al., 2006) were seeded on Transwells^®^ (24 mm diameter, 0.4 μm pore size, 4.67 cm^2^ culture surface area, Corning) at ~10^6^ cells/cm^2^. Parental Calu-3 cells were cultured in Eagle's minimum essential medium (EMEM) containing 15% fetal bovine serum (FBS), 1 mM sodium pyruvate and the non-essential amino acids glycine and L-isomers of alanine, asparagine aspartate, glutamate, proline, and serine at the concentrations normally used in MEM (Gibco, Burlington ON). CFTR KD cells were cultured in EMEM containing 7% FBS and 4 μg/ml puromycin. Any fluid that appeared spontaneously on the apical surface of monolayers after 1–2 days was removed to maintain the air interface. The basolateral medium was replaced with fresh medium every 2–3 days. After 2–3 weeks of culture in a humidified 5% CO_2_ incubator at 37°C, transepithelial resistance measured using an epithelial voltohmmeter (EVOM, World Precision Instruments, Sarasota FL) was >300 Ω cm^2^. The monolayer surface was rinsed with PBS and the basolateral medium was replaced with fresh Opti-MEM 1 day before assaying secretion. Monolayers were stimulated by adding forskolin (10 μM) to the basolateral medium. To collect secretions, inserts were tilted and the pipetor carefully pressed against the side of the dish to avoid contact with the cells. This manual method has been used previously to collect fluid repeatedly over several days (Shan et al., 2012). Fluid was secreted at a rate of ~40 μL/day for 2–3 days when sampled every 24 h. The constant secretion rate and development of a transepithelial bicarbonate gradient between secretions and basolateral fluid indicate that the sampling method did not cause significant leakage or contamination. Culture media and supplements were from Wisent (St. Bruno QC) except Opti-MEM, which was from Invitrogen (Burlington ON).

### Microtitrations

**Figure 1:**
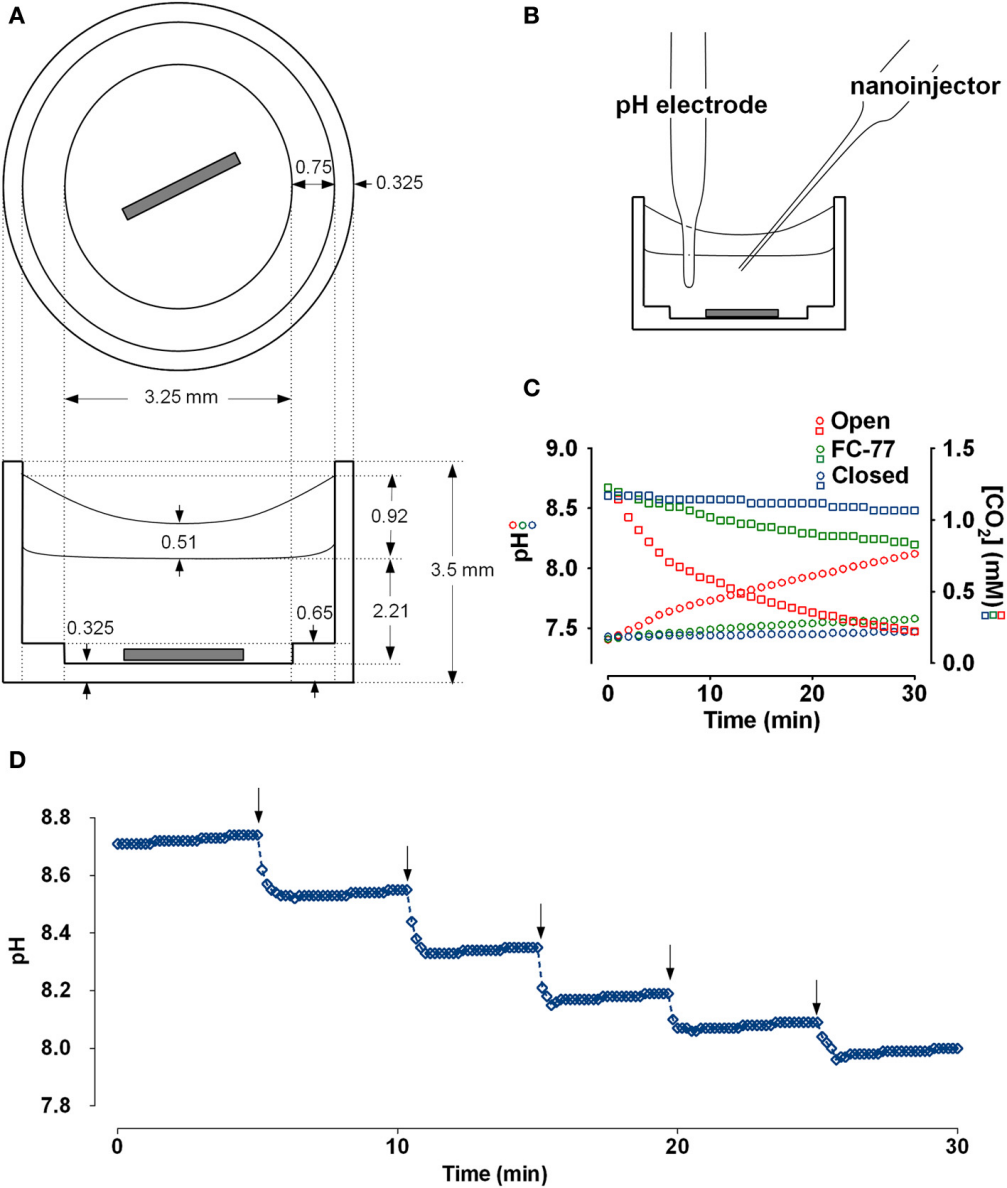
**Chamber schematic and characterization of the preparation. (A)** Top and side views of the polypropylene chamber. The sample (30 μl) was covered with a layer of FC-77 (10 μl) and stirred continuously. **(B)** Chamber arranged for microtitration. Micromanipulators holding the pH electrode and nanoinjector are not shown. **(C)** Measurement of CO_2_ efflux from the chamber under different conditions. An aliquot of 25 mM NaHCO_3_ solution (30 μl) that had been equilibrated with 5% CO_2_ was placed in the chamber and pH was measured at 1 min intervals. The red symbols show the increase in pH (circles) and decline in calculated CO_2_ concentration (squares) when sample was left open to the air. The green symbols show the pH (circles) and calculated [CO_2_] when sample was covered with 10 μl perfluorocarbon (FC-77). The blue symbols show the pH (circles) and calculated [CO_2_] when the chamber was completely immersed in a beaker of paraffin oil. **(D)** Time course of pH responses during a series of injections of 1N HCl using a Drummond Nanoject II (9.24 nl delivered per injection). pH stabilized within 1 min after adding titrant to the sample of Calu-3 secretions (30μl).

Samples from the apical surface of monolayers or from test solutions were divided into 2 aliquots. One was titrated with 1N HCl, the other with 1N NaOH, and results were combined to generate a complete titration curve. Aliquots (30 μl) of fluid were placed in a polypropylene chamber and mixed continuously using a stir bar which was fabricated from a stainless steel pin and driven by a magnetic stirrer (Figure 1A). Multiple cultures were sampled and pooled if required to obtain sufficient volume for analysis, however measurements were independent; i.e., fluid collected from a monolayer was not included in more than one sample. Ten μl of FC-77 (Sigma, vapor pressure 42 mmHg) was added onto the surface to minimize evaporation. Microtitrations were completed within ~20 min, before evaporation of the FC-77 (~30 min). pH was measured using a micro pH electrode (Orion) positioned in the sample using a micromanipulator. A calibrated Drummond Nanoject II injector, also positioned with a micromanipulator, was used to deliver titrant in steps of 9.24 nl (Figure 1B). The upper surface area covered by FC-77 was 0.177 cm^2^. The total area of the sample including surfaces in contact with the chamber was 0.684 cm^2^.

**Figure 2:**
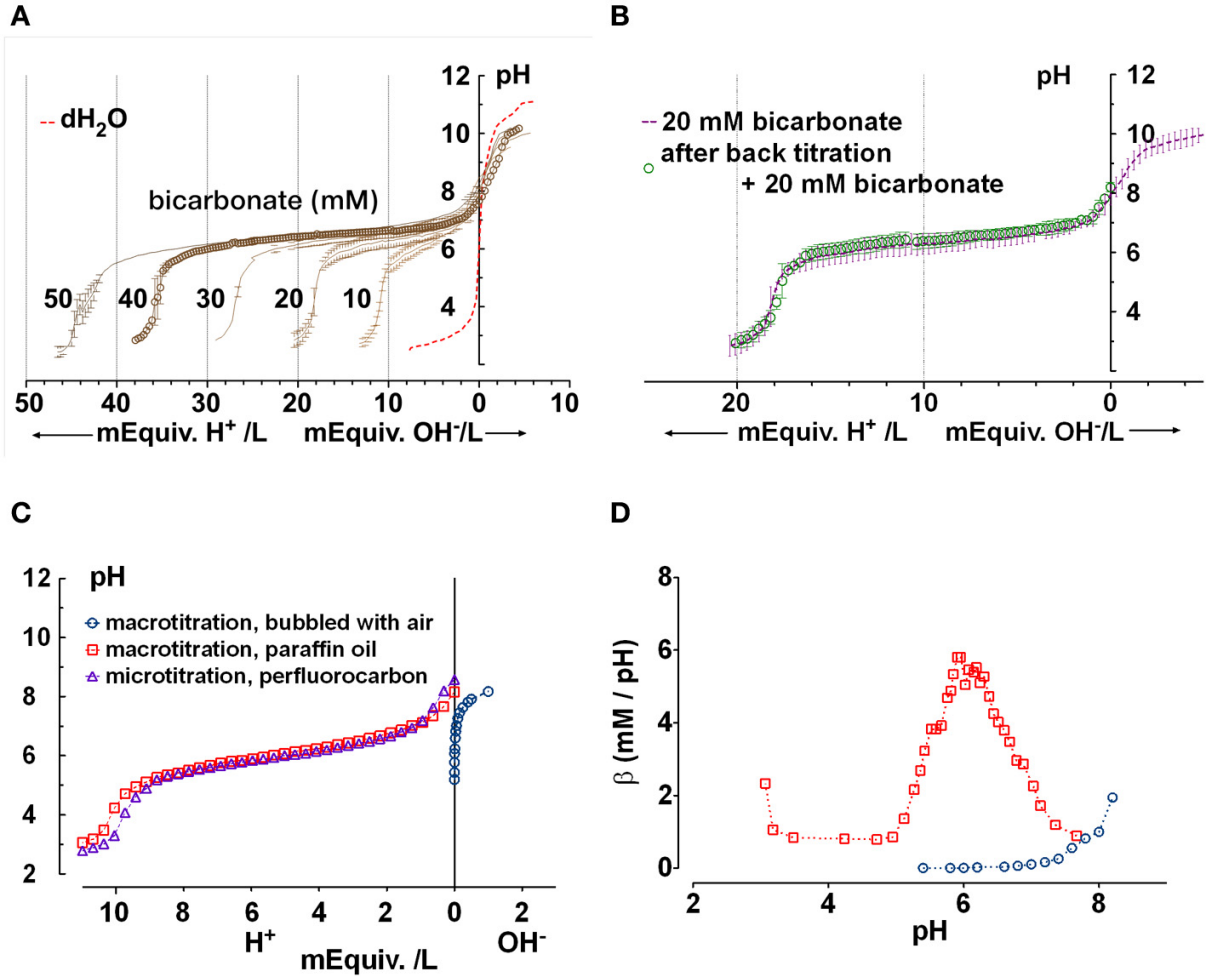
**Confirmation that the chamber approximates a closed system for CO_2_/HCO^−^_3_. (A)** Microtitration curves obtained for pure solutions having sodium HCO^−^_3_ concentrations between 10 and 50 mM. Unbuffered water is shown for comparison (dashed red line). Means ± SD, *n* = 3–5. **(B)** Reproducibility of microtitrations. A sample of 20 mM HCO^−^_3_ solution was microtitrated with HCl, neutralized using NaOH, supplemented with 20 mM HCO^−^_3_ by injection of concentrated HCO^−^_3_ solution, and re-titrated. The second titration curve (open circles) was within the standard deviation of the initial curve. Means ± SD, *n* = 3–5. **(C)** Comparison of (Δ) microtitration curve for 10 mM NaHCO_3_ solution when samples are covered by perfluorocarbon and (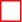) macrotitration of the same solution performed in a closed under paraffin oil. Also shown is a titration curve for water titrated with NaOH in an open system (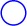, i.e., vigorously bubbled with air). The microtitration results resemble those obtained by macrotitration under paraffin oil (i.e., a closed system). **(D)** equilibrium buffer capacity calculated over a similar pH range from macroscopic titrations under open (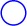, bubbled with air) vs. closed conditions (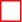, under paraffin oil).

To measure CO_2_ efflux, 30 μl of 25 mM NaHCO_3_ solution that had been pre-equilibrated with 5% CO_2_ was placed in the chamber along with the stirring pin, pH electrode, and nanoinjector syringe. CO_2_ loss was monitored by measuring the pH at 1 min intervals with continuous stirring. CO_2_ concentration was calculated using the Henderson-Hasselbalch equation by assuming equilibration and a CO_2_ solubility coefficient of 0.03 mM/mmHg. The time course of pH and calculated dissolved [CO_2_] under different conditions are shown in Figure 1C. During the first 5 min, 13.8 nmoles of CO_2_ were lost from the sample (red squares). The first 6 data points (at *t* = 0, 1, 2, 3, 4, 5 min) were used to calculate the initial rate of CO_2_ efflux from the sample. With an initial P_CO2_ gradient between the sample and atmosphere of 40.75 mmHg and total sample area of 0.6842 cm^2^, we calculated a CO_2_ permeability constant for the system of 0.167 × 10^−6^ cc at standard temperature and pressure/cm^2^/s/10 mmHg. Based on the published permeability constant for polypropylene (9.2 × 10^−9^ cc @ STP/cm^2^/mm/s/10 mmHg) this suggests that ~8.4% of the CO_2_ efflux occurred through the walls of the polypropylene chamber and the other 91.6% escaped from the top surface of the sample. The same procedure was repeated after covering the sample with a layer of 10 μl FC-77. The initial rate of decline of the CO_2_ was greatly reduced with the perfluorocarbon (PFC) layer and appeared more linear (green squares in Figure 1C), therefore we used the first 12 points to calculate the initial CO_2_ efflux rate and obtained a permeability constant of 2.78 × 10^−8^ cc @ STP/cm^2^/s/10 mmHg; i.e., 16.6% of the CO_2_ permeability of the open chamber. Thus the layer of FC-77 reduced the rate of CO_2_ loss by ~83%. Finally, the chamber, pH electrode, and nanoinjector syringe were all submerged in a beaker of paraffin oil and the rise in pH was again measured at 1 min intervals with stirring. Under these conditions the pH increased by ~0.1 units after 0.5 h and the calculated CO_2_ concentration declined by 9.36%. All the points were used to calculate the rate, which yielded the permeability constant 0.686 × 10^−8^ cc @ STP/cm^2^/s/10 mmHg, or 24.3-fold lower than when the chamber was open. These measurements of CO_2_ permeability indicate the microtitration system is ~6-fold closer to a closed than an open system. This was further confirmed by comparing microtitration curves for a pure 20 mM HCO^−^_3_ solution with those obtained by macroscopic titration under a layer of paraffin, or when bubbled with air without paraffin oil (i.e., closed vs. open system; see below and Figure 2C).

CO_2_ or HCO^−^_3_ concentration was calculated using the Henderson-Hasselbalch equation:

(1) $$\text{pH = pK +}{\text{log}}_{10}\frac{\left[{\text{HCO}}_{3}^{-}\right]}{s\;\cdot {\text{P}}_{{\text{CO}}_{2}}}$$

where pH and pK have their usual meanings, *s* is the solubility of CO_2_, and P_CO2_ is the partial pressure of CO_2_ above the sample. Buffer capacity (or buffer value) β was calculated as a function of pH using the slope of the microtitration curve; i.e., the first derivative

(2) $$\beta =\;\frac{\Delta \text{C}}{\left|\Delta \text{pH}\right|}$$

where ΔC is the increase in H^+^ or OH^−^ concentration in the sample and ΔpH is the resulting pH change (Van Slyke, 1922). Buffer capacity in a closed system is

(3) $$\beta =2.3\;\cdot \;\left[\text{TB}\right]\frac{\left[{\text{H}}^{+}\right]\;\cdot \text{ K}}{{\left(\left[{\text{H}}^{+}\right]\text{+ K}\right)}^{2}}$$

where [TB] is the total buffer concentration and K is the equilibrium constant. When plotted as a function of pH this expression yields a bell shaped curve with a peak buffer capacity of 0.58·[HCO^−^_3_]. In an open system, β simplifies to

(4) $$\beta =\;2.3\;\cdot \;[{\text{HCO}}_{\text{3}}^{-}]$$

and is predicted to increase exponentially with pH (Roos and Boron, 1981).

pH was measured ~1 min after the addition of titrant by which time the pH was stable as illustrated in Figure 1D, which shows the titration of Calu-3 fluid using a longer time interval (5 min) between acid injections. Adding the carbonic anhydrase inhibitor acetazolamide (100 μM) to samples did not affect the rate of pH equilibration noticeably (data not shown) and previous proteomics studies have not detected carbonic anhydrase in airway secretions (Candiano et al., 2007). Thus CO_2_ hydration and dehydration reactions during microtitrations probably occurred at their uncatalyzed rates.

### Composition of secreted fluid

Secretions from stimulated Calu-3 monolayers and basolateral medium were analyzed for electrolytes using a Beckman Coulter UniCel DxC 800 Synchron Clinical System (see Supplementary Materials for details). Anion gap (AGAP) was calculated as (Na^+^ + K^+^ + 2Ca^2+^ + 2Mg^2+^) – (Cl^−^ + HCO^−^_3_) to check for metabolic acid production. Total protein was measured using the Bradford assay. Lysozyme was quantified using an Enzyme-Linked Immunosorbent Assay (ELISA) kit according to the manufacturer's protocol (Biomedical Technologies Inc., Stoughton, MA).

### Immunoblotting

Samples were subjected to SDS-PAGE on 12% gels. Proteins were transferred to nitrocellulose membranes (Luo et al., 2009) and probed with sheep anti-human lysozyme antibody (1:100; Biomedical Technologies, Inc.). Blots were washed, incubated with a secondary antibody that had been conjugated to horseradish peroxidise (1:1000) and visualized by enhanced chemiluminescence (Amersham Biosciences, Baie d'Urfé, QC). Blots were scanned and analyzed using ImageJ (Rasband, 2011).

### Statistics

Transepithelial ion concentration gradients and differences in volatile buffer concentration were evaluated using Student's unpaired *t*-tests, with *p* < 0.05 considered significant.

## Results

### Microtitration of pure HCO^−^_3_ solutions suggest a closed system

We began by determining microtitration curves using known HCO^−^_3_ concentrations as standards, to confirm that the titration conditions approximate a closed system in which CO_2_ remains in solution rather than an open system, which allows CO_2_ equilibration with the air.

Microtitration of pure solutions containing 0, 10, 20, 30, 40, and 50 mM HCO^−^_3_ yielded the series of curves shown in Figure 2A. The apparent pK_a_ (6.34 ± 0.14) was higher than the usual value quoted of 6.1, which may be due to finite CO_2_ leakage and/or low ionic strength (0.01–0.05 M vs. the standard 0.15 M). Nevertheless, the results were reproducible as demonstrated when the forward microtitration was repeated using the same sample after it had been alkalinized with NaOH and 20 mM HCO^−^_3_ had been restored by injection of ~600 nl of 1 M NaHCO_3_ solution. This procedure, which increased the 30 μl volume by 2%, yielded data points within the standard error of the original curve (Figure 2B).

To further confirm that the microtitration preparation approximates a closed system, we compared the microtitration of 10 mM NaHCO_3_ with macroscopic titrations performed under paraffin oil (i.e., in a closed system), or without oil while vigorously bubbling with air (open system; Figure 2C). The microtitration curve closely resembled the macroscopic curve obtained under paraffin oil (i.e., the closed system). When the slope of the titration curve was calculated at different pHs using a 5 point window, the peak β under paraffin oil was 6.4 mM/pH, in reasonable agreement with the theoretical buffer capacity for a perfectly closed system (5.8 mM/pH) (Figure 2D). When the macroscopic titration was performed by the stepwise addition of NaOH to water with vigorous bubbling, the β calculated after equilibration with prolonged aeration increased exponentially. These results confirm that the microtitration conditions approximate those for a closed system.

### Buffer capacity of Calu-3 secretions under basal and cAMP-stimulated conditions

**Figure 3:**
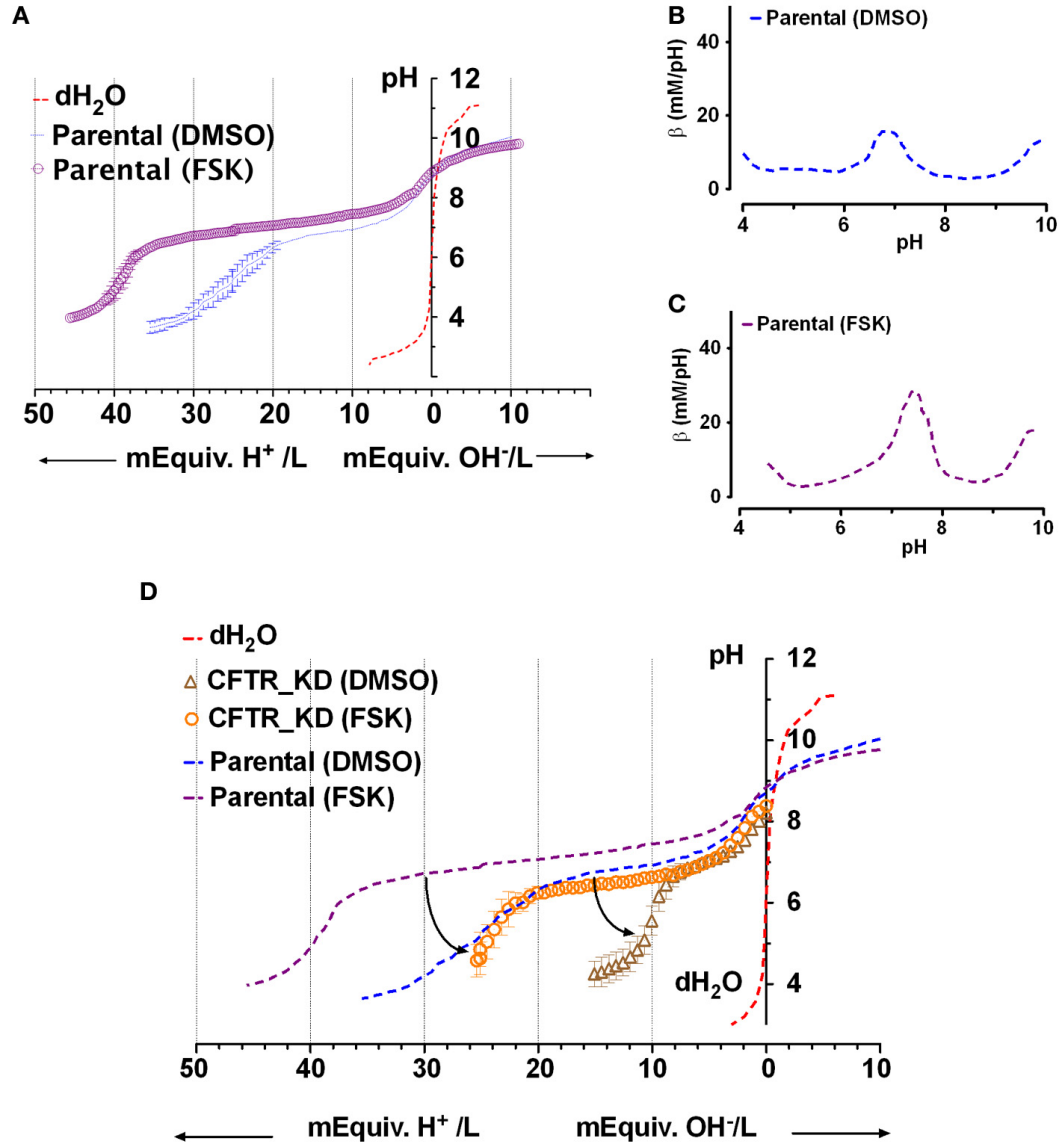
**Titration curves for Calu-3 secretions. (A)** Microtitration of fluid produced by parental Calu-3 cell monolayers under basal conditions (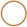, treated with DMSO vehicle) and during stimulation by 10 μM forskolin (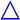). Dotted line shows the control curve obtained with distilled water. HCl and NaOH were the titrants and were added in increments of 0.4 mM final concentration. Means ± SD, *n* = 4–5. **(B,C)** Mean buffer capacity β for fluid from unstimulated (DMSO) and forskolin (FSK) stimulated Calu-3 secretions, respectively, calculated from **(A)**. **(D)** Effect of CFTR knockdown on the titration curves obtained under basal conditions (DMSO) and during forskolin stimulation (FSK). Curved arrows show the impact of CFTR on buffering of Calu-3 secretions.

Figure 3A shows mean titration curves obtained for fluid secreted by parental Calu-3 monolayers under basal conditions (DMSO vehicle control) and during stimulation by 10 μM forskolin. Control curves without buffer (distilled water, dashed red line) are also shown for comparison. Secretions initially had high pH (8.9 ± 0.3, *n* = 4) when placed in the chamber, indicating partial equilibration with the low P_CO2_ of room air. The initial pH of distilled water was moderately acidic (pH 6) as expected. Inspection of the curves indicates that most buffering of the secreted fluid occurred in the range pH 6–8. When inflection points were determined using Prism 5 software, the fluid from unstimulated Calu-3 cells was most strongly buffered between pH 7.64 and 6.01, the two nearest stationary points in the second derivative curve. Within this range β was 13 ± 7 mM/pH (*n* = 4).

Forskolin stimulation increased the buffer capacity to 24 ± 5 mM/pH (*n* = 4). Figures 3B,C compare the mean β as a function of pH for unstimulated vs. forskolin-stimulated monolayers, respectively. β displayed a central peak under both conditions, which was ~0.5 units more alkaline than the pK_a_ for pure HCO^−^_3_ solutions. Comparison of Figures 3A and 2A suggests that forskolin stimulation raises the HCO^−^_3_ concentration of Calu-3 secretions from ~24 to 44 mM and may cause the release of another buffer.

### Effect of CFTR knockdown on the buffer capacity of secretions

To assess the role of CFTR in HCO^−^_3_ secretion, microtitrations were performed using fluid from CFTR knockdown cells ± forskolin (Figure 3D). Secretions from CFTR KD cells (pH 8.2 ± 0.5, *n* = 3) were less alkaline than from control cells (8.9 ± 0.3), and β between pH 6.4 and 7.6 (the stationary points of the second derivative curve) was reduced to 5 ± 1 mM/pH. Forskolin still increased β more than 2-fold to 11 ± 4 mM/pH in secretions from CFTR KD cells, presumably due to stimulation of residual CFTR that was expressed despite shRNA knockdown (<5% of parental cells). HCO^−^_3_ concentration was reduced by more than half compared to the fluid secreted by parental cells, ranging from 10 mM under basal conditions to 25 mM during forskolin stimulation. These results indicate that CFTR plays a major role in HCO^−^_3_ secretion by Calu-3 cells under these non-pH stat conditions.

### Evidence for other buffers

**Figure 4:**
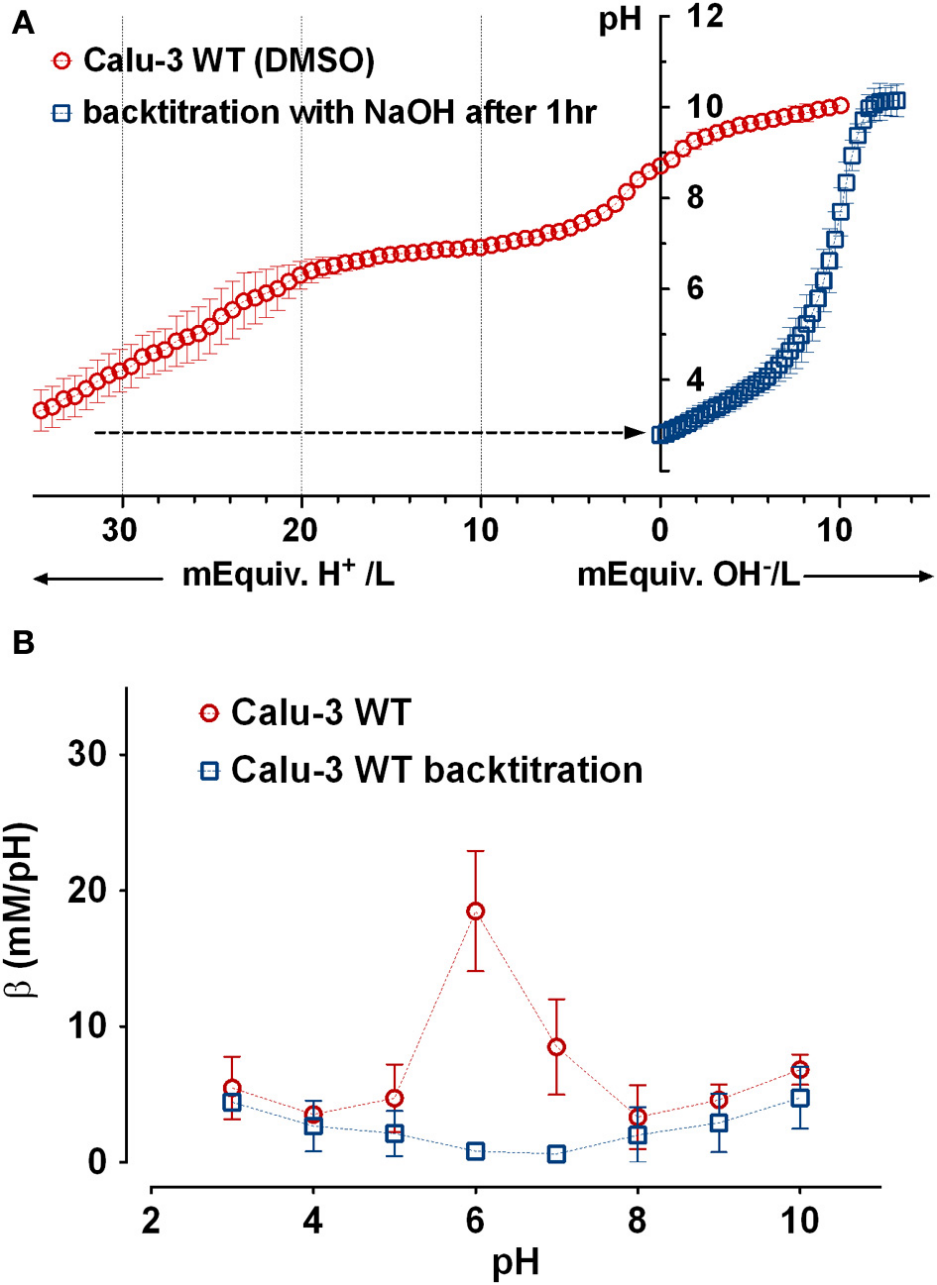
**Evidence for a non-bicarbonate buffer in Calu-3 fluid. (A)** Forward and back titration curves for Calu-3 secretions. Samples were equilibrated with air for 1 h at pH < 4 after the forward titration to drive off CO_2_. **(B)** Buffer capacity as a function of pH calculated from the forward (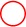) and back (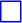) titrations. Means ± SD; *n* = 3–5.

To confirm that most buffering of Calu-3 secretions was due to HCO^−^_3_ and test for the presence of non-HCO^−^_3_ buffers, Calu-3 fluid was collected, titrated to pH 4 with HCl, equilibrated with room air CO_2_ for 1 h to release any CO_2_ generated, then back titrated to the starting pH with NaOH (Figure 4A). Approximately 3-fold more acid than base equivalents were consumed during the forward and back titrations, respectively, and the large peak in β near pH 6 during the forward titration was absent from the back titration curve (Figure 4B, Table 2). The β attributable to non-volatile buffers had a U-shaped dependence on pH and was lowest near the pKa for carbonic acid.

### Composition of the secretions

Secretions were analyzed for electrolytes and metabolic acid production. Sodium, potassium, chloride, HCO^−^_3_, albumin, calcium, magnesium, and total protein were measured in Calu-3 secretions and are shown in Table 1 along with the calculated anion gap ([Na^+^] + [K^+^] + 2[Ca^2+^] +2[Mg^2+^]) − ([Cl^−^] + [HCO^−^_3_]) for comparison with the basolateral medium. Chemical analyses yielded a lower HCO^−^_3_ concentration (35.7 mM) than did microtitrations (44.6 mM), nevertheless the [HCO^−^_3_] was >2.5-fold higher in secretions than in the basolateral medium, and this elevation was partially offset by lower apical [Cl^−^]. K^+^ and Mg^2+^ were lower in the secretions whereas Ca^2+^ was slightly higher. Forskolin stimulation for 24 h did not alter the basolateral composition significantly or cause a large increase in the anion gap, therefore a high rate of secretion did not result in the production of large amounts of metabolic acid. To examine their potential contributions to β, microtitrations were also performed using individual components at concentrations relevant to Calu-3 fluid or reported for ASL *in vivo*.

**Table 1:** Analysis of Calu-3 secretions and basolateral media^*^.

	**AP secretions with FSK**	**BL medium with FSK**	**BL medium**
**Sodium (mM)**	152.7 ± 6.7	145.7 ± 1.2	145.0 ± 1.0
**Potassium (mM)**	4.8 ± 0.6^‡^	6.2 ± 0.1	5.9 ± 0.1
**Chloride (mM)**	119.0 ± 8.9	132.0 ± 0	129.7 ± 0.6
**Bicarbonate (mM)**	35.7 ± 1.5^‡^	13.0 ± 1.7	13.0 ± 1.0
**Calcium (mM)**	2.2 ± 0.1^‡^	1.9 ± 0.1	1.9 ± 0.01
**Magnesium (mM)**	0.54 ± 0.1^‡^	0.9 ± 0.04	0.82 ± 0.01
**AGAP (mM)**	5.6 ± 4.2	9.7 ± 0.4	11 ± 0.5

Analysis of Calu-3 apical (AP) secretions, and basolateral (BL) medium from cultures treated with forskolin (FSK) for 24 h, for comparison with medium before exposure to cells (n = 3).

* Mean ± SEM; n=3;

‡ p < 0.05.

**Table 2:** pH-dependent buffer capacity (β) of forskolin-stimulated Calu-3 secretions before and after neutralizing bicarbonate^*^.

	**pH 3–4**	**pH 4–5**	**pH 5–6**	**pH 6–7**	**pH 7–8**	**pH 8–9**	**pH 9–10**	**>pH 10**
**Forward titration**	5.5 ± 2.3	3.5 ± 0.5	4.7 ± 2.4	18.5 ± 4.4^‡^	8.5 ± 3.5^‡^	3.3 ± 2.3	4.6 ± 3.4	6.8 ± 1.1
**Back titration**	4.4 ± 0.7	2.6 ± 1.8	2.1 ± 1.6	0.8 ± 0.2	0.6 ± 0.5	2.0 ± 2.0	2.9 ± 2.2	4.8 ± 2.3
**% of *p* due to HCO^−^_3_**	19	24	55	96	93	40	37	30

* mM/pH; % of β due to HCO^−^_3_ = 100*((β-pback)/β); Mean ± SD; n=4;

‡ p < 0.05

### Phosphate

**Figure 5:**
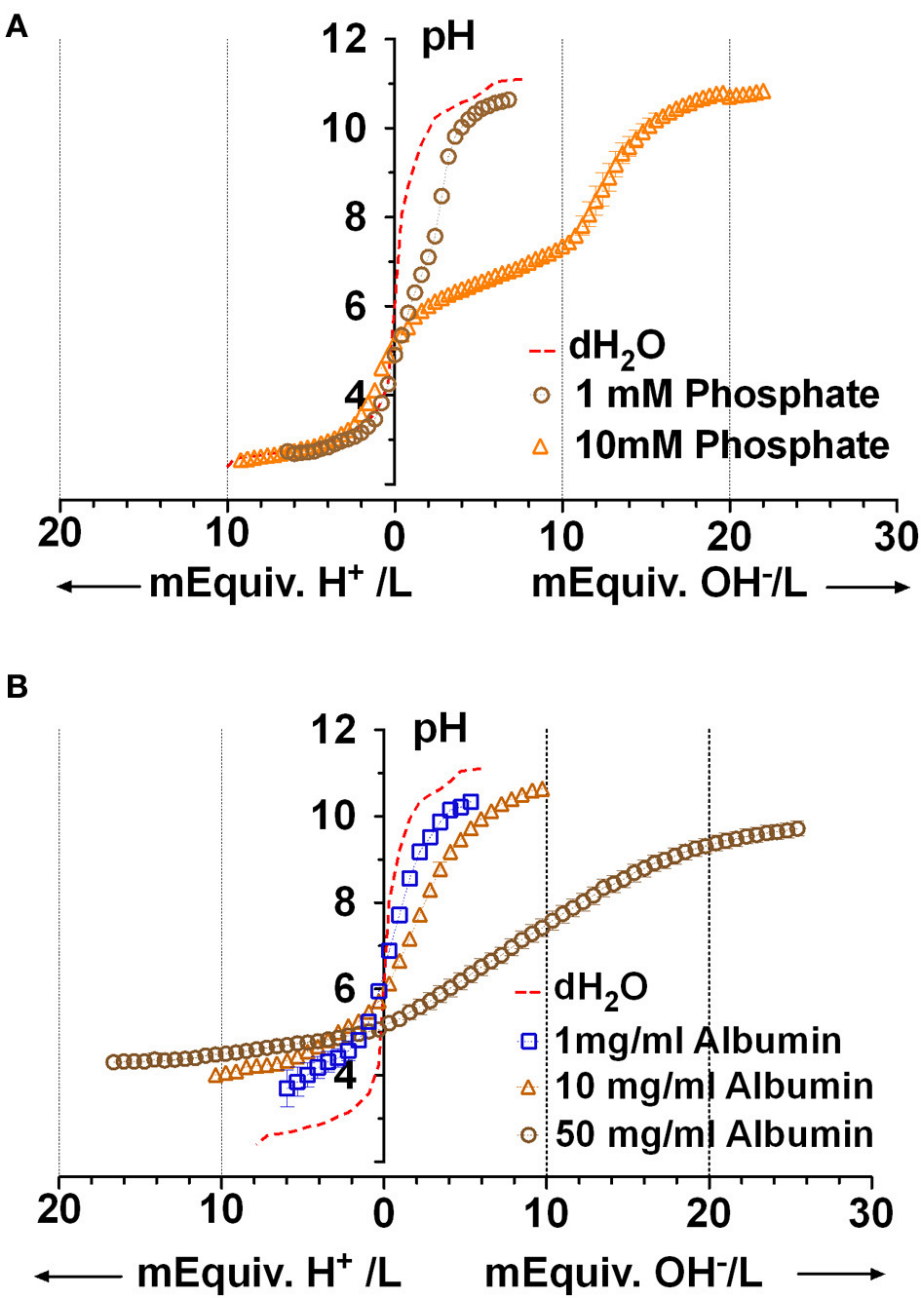
**Microtitration curves for phosphate and albumin at relevant concentrations. (A)** Curves obtained with 1 and 10 mM phosphate solutions. Dashed red line shows results with distilled water. **(B)** Microtitration curves obtained with albumin. Means ± SD; *n* = 4.

Although the PO^=^_4_ concentration in Calu-3 secretions was below the detection limit of the Beckman Coulter UniCel DxC 800 Synchron Clinical System, millimolar PO^=^_4_ levels have been measured in mouse ASL using capillary electrophoresis (1.9 mM; Govindaraju et al., 1997). Therefore we performed microtitrations to assess the possible impact of 1 and 10 mM KH_2_PO_4_ on Calu-3 fluid. The three pK_a_s of phosphoric acid were easily resolved with 10 mM KH_2_PO_4_ solution (Figure 5A). The buffer capacity of 10 mM PO^=^_4_ between pH 7.56 and 5.53 was 5.1 ± 1.4 mM/pH (*n* = 4). However β could not be measured accurately with 1 mM KH_2_PO_4_ solution. Since the β for 10 mM PO^=^_4_ solution was low compared to Calu-3 secretions under closed conditions, its contribution in the ASL would also be small relative to HCO^−^_3_.

### Proteins

Protein is released into the airway lumen passively (exudates), by transepithelial transport (Webber and Widdicombe, 1989), and can be synthesized and released by submucosal gland acinar cells (Jacquot et al., 1988). Airway secretions, which were reported to contain “albumin-like protein” (Jacquot et al., 1988), have more recently been found to comprise ~175 proteins. To estimate the buffer capacity that may be mediated by proteins we used albumin as a model protein. Using the Bradford assay we measured 7.4 ± 2.3 mg protein/ml in Calu-3 secretions under basal conditions and 5.8 ± 1.5 mg/ml during forskolin stimulation (means ± S.E., *n* = 5). Forskolin stimulated the rate of fluid secretion rate by ~8-fold and reduced the protein concentration in secretions by 22%, suggesting a >7-fold stimulation of protein secretion. Microtitration of 1, 10, and 50 mg/ml albumin solutions revealed buffering over a wide range which was strongest at extreme pH; i.e., below pH 5 (β = 10.6 ± 4.4 mM/pH with 50 mg/ml, *n* = 4) and above pH 9 (β = 3.9 ± 1.1 mM/pH, *n* = 4 with 50 mg/ml; Figure 5B).

### Lysozyme

**Figure 6:**
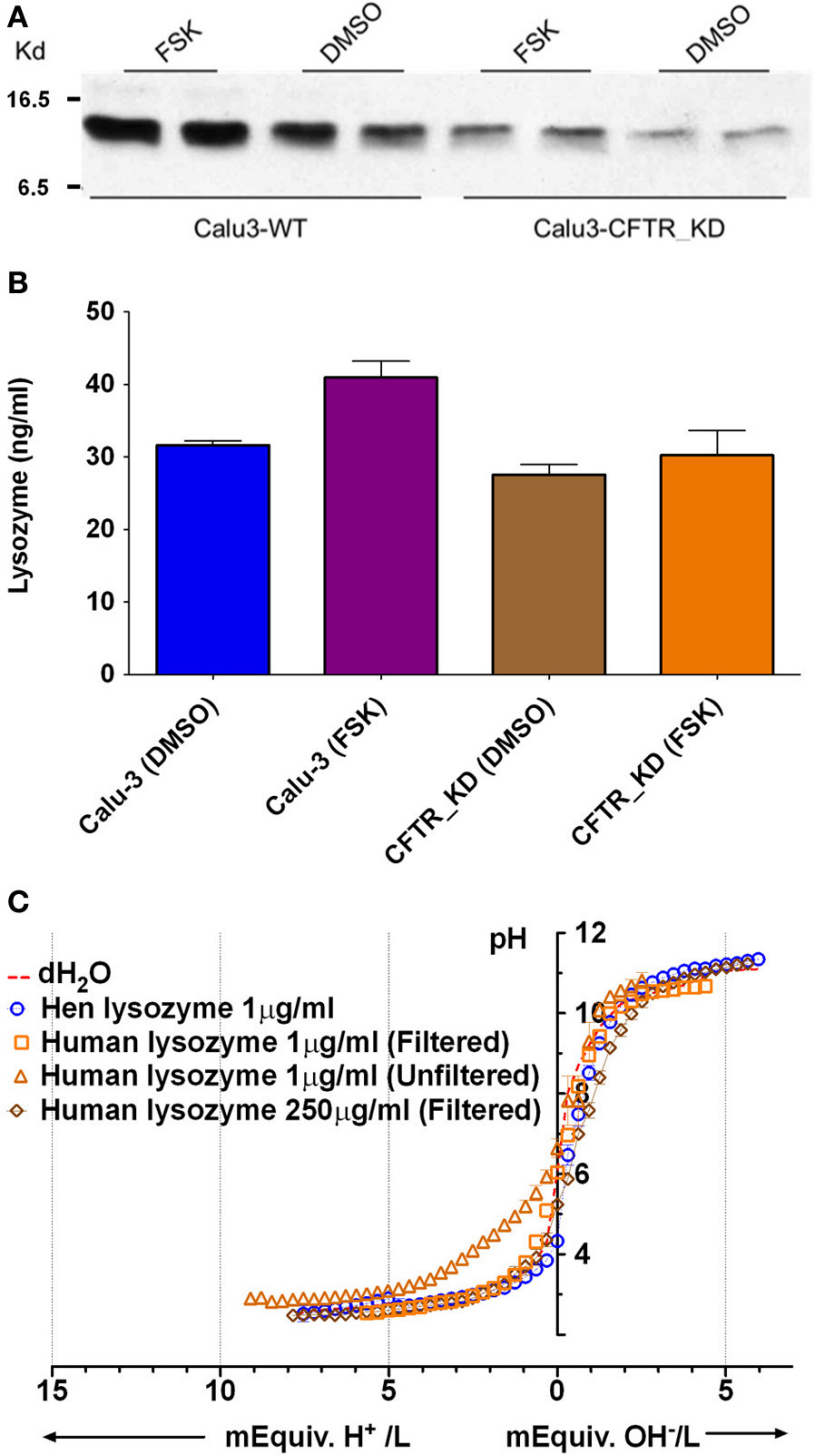
**Lysozyme expression and buffer capacity in pure solutions. (A)** Immunoblot probed with anti-lysozyme antibody in duplicate samples from control Calu-3 and Calu-3 CFTR KD secretions under basal conditions and during forskolin stimulation. **(B)** Quantitation of lysozyme by ELISA under each condition shown in **(A)**. Data are means ± SD; *n* = 3. **(C)** Titration curves for lysozyme solutions (*n* = 4). Dotted line also shows the pH of distilled water as a reference (unbuffered). Some aliquots of lysozyme were desalted by filtration to remove sodium acetate.

Lysozyme is produced by submucosal glands and by Calu-3 cells, and it is relatively abundant in ASL (Duszyk, 2001; Dubin et al., 2004; Joo et al., 2004) and has maximal β near pH 4 (Olthuis et al., 1994). Immunoblots confirmed that both parental and CFTR KD Calu-3 cells release lysozyme (Figure 6A). ELISAs revealed a 30% increase in the lysozyme concentration in secretions from parental Calu-3 cells (control 31 ± 1.1 ng/ml, forskolin 43 ± 3.2 ng/ml; *p* < 0.05, *n* = 3; Figure 6B). As shown above for total protein, these results imply a 10.4-fold increase in total lysozyme release during stimulation. Although lysozyme concentration is too low to buffer Calu-3 fluid significantly, there are 32 ionizable groups per lysozyme molecule and >2000-fold higher concentrations are expected *in vivo*, therefore we tested solutions that contained up to 250 μg/ml. Lysozyme did not buffer significantly at the highest concentration after it had been desalted to remove sodium acetate (cutoff *M*_*r*_ = 2 kD, lysozyme *M*_*r*_ = 14.6 kD) to (Figure 6C). These results indicate that lysozyme contributes little to the buffer capacity of Calu-3 secretions or ASL *in vivo*.

### Mucins

**Figure 7:**
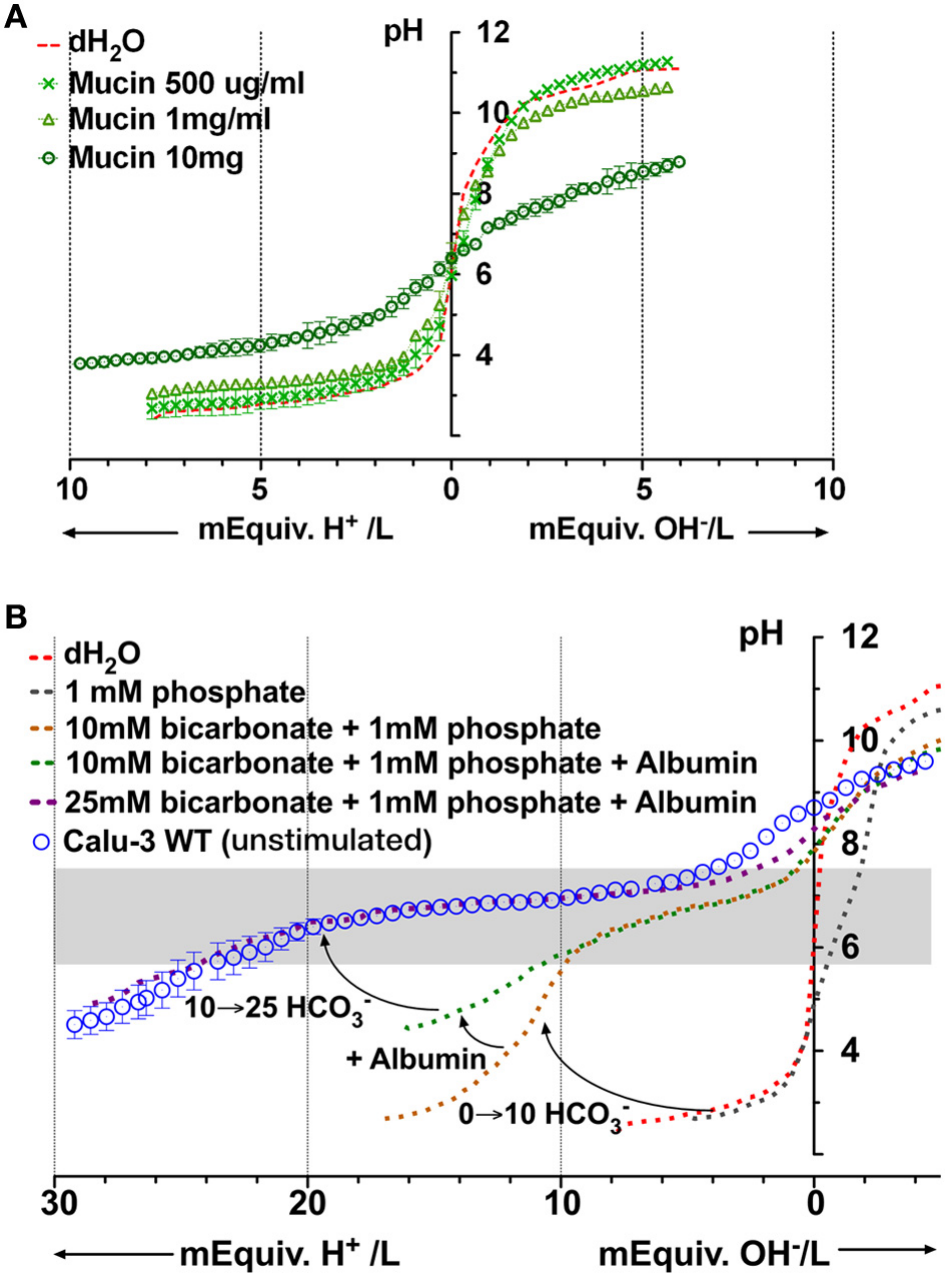
**Microtitration curves for porcine gastric mucins and simulation of Calu-3 fluid buffer capacity using individual components. (A)** Buffering by different concentrations of mucin. **(B)** Simulation of Calu-3 by mixtures of PO^=^_4_, HCO^−^_3_, and albumin. Curved arrows show the effect of adding 10 mM HCO^−^_3_, 10 mg/ml albumin, and raising the concentration of HCO^−^_3_ to 25 mM. The gray rectangle represents the region between the stationary points of the buffer.

Mucins are produced by submucosal glands and also by Calu-3 cells (Dubin et al., 2004; Kreda et al., 2007; Lesimple et al., 2013). Unstimulated Calu-3 cells secrete these acidic glycoproteins at a rate of ~300 ng/cm^2^ per day (Dubin et al., 2004) and fluid at the rate of ~7.5 μl/cm^2^ day, which together predict a final concentration of ~40 μg/ml. We performed microtitrations with porcine gastric mucins, which are similar to those secreted by the airways. MUC5AC is the most abundant isoform pig gastric mucin, followed by MUC2, MUC5B, and MUC6 (Caldara et al., 2012). Porcine gastric mucins did not buffer significantly at concentrations up to 1 mg/ml (Figure 7A), therefore mucins apparently contribute little to the buffer capacity of Calu-3 secretions. However *in vivo* the airway mucus is typically 2–3% solids (20–30 mg/ml), and dissolved mucins may reach ~12 mg/ml in the periciliary liquid, the concentration at which they undergo a sol-gel transition (McCullagh et al., 1995). When similar high concentrations of pig gastric mucins (e.g., 10 mg/ml) were titrated we obtained a β of ~5 mM/pH. Although mucins contributed little buffer capacity to Calu-3 secretions, these results suggest they may contribute significantly to ASL buffering *in vivo*.

### A model fluid to simulate buffering of Calu-3 secretions

Based on the preceding results we tried to simulate the titration curve for Calu-3 secretions using defined components. PO^=^_4_ contributed minimally to β in Calu-3 secretions but was included to weakly stabilize the pH, facilitating measurement of the control curve. Figure 7B shows the curves obtained for solutions containing PO^=^_4_ alone, PO^=^_4_ + HCO^−^_3_, and PO^=^_4_ + HCO^−^_3_ + albumin. The combination of 1 mM PO^=^_4_ and 10 mM HCO^−^_3_ provided insufficient buffering below pH 6 and the shape of the curve was different from that for Calu-3 secretions. However the β at low pH (pH < 6) increased when albumin (10 mg/ml) was added. When [HCO^−^_3_] was raised further to 25 mM as estimated in basal Calu-3 secretions, the titration curve for the defined solution closely resembled that for fluid from unstimulated Calu-3 monolayers (Figure 7B). Thus it was possible to mimic the titration curve for Calu-3 fluid with a solution containing HCO^−^_3_, albumin, and a low concentration of PO^=^_4_. The β between stationary points at pH 7.72 and pH 5.88 for the model solution was 19 ± 2.3 mM/pH (*n* = 4) and buffering was maximal at pH 7; i.e., near the normal pH of ASL according to most reports (Fischer and Widdicombe, 2006).

## Discussion

In this study we developed a method for titrating micro-scale volumes (30 μl) and used it to study fluid secreted by the human airway cell line Calu-3. The assay behaved like a closed system due to a covering layer of PFC, which was necessary to minimize evaporation of the small sample volume. The rate of CO_2_ diffusion in a related PFC perfluorobutyltetrahydrofurane (FC-80) is ~2500-fold slower than in air (Schoenfisch and Kylstra, 1973). Thus adding a layer of PFC would be expected to slow CO_2_ efflux from the sample, and this was observed experimentally.

CO_2_ solubility is 3-fold higher in PFC than in water however its CO_2_ diffusion coefficient is similar in both solvents. High CO_2_ solubility in PFC would increase the rate of CO_2_ diffusion from the PFC layer to the atmosphere but would reduce it from the sample into the PFC layer. The steady state efflux of CO_2_ from the sample would depend on the gradient between the sample and the atmosphere, therefore partitioning into the PFC should have little effect on CO_2_ loss. Microtitrating pure NaHCO_3_ solutions yielded β close to the theoretical value for a closed system (0.58 × [total buffer]). The β values for 10, 20, 30, 40, and 50 mM NaHCO_3_ were 5.7, 13.1, 18.9, 25.3, and 33.2 mM/pH respectively, and the stationary points were 5.2 and 7.2 for all five concentrations. Buffer capacity calculated from control macroscopic titrations followed predictions under both closed and open systems, however pH equilibration was very slow in the open system, perhaps due to the high pH when titrating air-equilibrated bicarbonate solutions. CO_2_ produced immediately after the addition of HCl may have been converted back to bicarbonate by the relatively fast hydroxylation reaction CO_2_ + OH^−^ ↔ HCO^−^_3_, which would slow CO_2_ release from the solution despite vigorous bubbling. Computer simulations would be useful for testing this quantitatively.

Lysozyme and mucins contributed little to the buffer capacity of Calu-3 fluid due to their low concentrations, however buffering by proteins was significant. Interestingly, forskolin stimulated fluid secretion rate by ~8-fold whereas protein concentration only declined from 7.4 to 5.8 mg/ml, indicating that cAMP may also stimulate protein secretion by >7-fold. There is longstanding evidence for a protein secretion defect in CF exocrine glands (McPherson et al., 1986), and CFTR deficient Calu-3 cells might be useful for studying the mechanism. Titrations performed with porcine gastric mucins over a range of concentrations suggest that mucins may also buffer ASL significantly *in vivo*.

HCO^−^_3_ is the main pH buffer in blood and many other fluids. Calu-3 cells secrete HCO^−^_3_ (Lee et al., 1998; Devor et al., 1999; Shan et al., 2012), and β increased from 13 to 24 mM/pH during forskolin stimulation, consistent with an ~2-fold increase in [HCO^−^_3_]. The basal [HCO^−^_3_] estimated by microtitration (22.4 mM) was in reasonable agreement with that reported for parental Calu-3 cells under similar conditions (26 mM; Garnett et al., 2011) but lower than a virtual gland preparation in which secretions exit through a small opening that mimics a duct (36 mM; Irokawa et al., 2004). The virtual gland preparation may allow CO_2_ and HCO^−^_3_ to accumulate to higher levels than open Transwells^®^. The present study suggests that [HCO^−^_3_] was increased >2-fold during forskolin stimulation, although they did not reach the levels reported previously (60–74 mM HCO^−^_3_). These discrepancies may reflect differences in sample handling or in CFTR expression. Regardless, there is general consensus that secretions are 0.3–0.4 units more alkaline during forskolin stimulation than under basal conditions.

The role of CFTR in HCO^−^_3_ secretion by Calu-3 cells is presently under debate (Devor et al., 1999; Garnett et al., 2011; Shan et al., 2012). The present finding that β is reduced by half in CFTR KD fluid indicates that CFTR plays an important role in HCO^−^_3_ secretion, and is consistent with the acidic secretions produced by CF glands compared to control glands (Song et al., 2006). However another group found no difference in the pH of secretions produced by CFTR-KD vs. control Calu-3 cells (Garnett et al., 2011). The reason for the different results is not known, but may reflect the higher level of residual CFTR expression in the knockdown cells used in the latter study. Although the CFTR blocker GlyH101 (10 μM) also did not reduce the pH or [HCO^−^_3_] of secretions consistent with the CFTR knockdown result, ~45% of the fluid secretion persisted during GlyH101exposure, suggesting CFTR may not have been fully inhibited during the 24 h assay. It has been shown that CFTR inhibition by another antagonist (CFTR^inh^-172, 5 μM) declines by 50% within the first 6 h during long-term experiments (Perez et al., 2007). In the present study, microtitration of fluid from CFTR KD monolayers indicated that CFTR is important for both basal and forskolin-stimulated HCO^−^_3_ secretion, in agreement with pH-stat studies (Shan et al., 2012).

Approximately 175 proteins have been detected in bronchoalveolar lavage fluid and in secretions from epithelial cultures *in vitro* (Magi et al., 2002; Candiano et al., 2007; Ali et al., 2011). Proteins are significant pH buffers in plasma and other extracellular fluids, therefore we examined their contribution to β in Calu-3 fluid. Calu-3 secretions contained 5–7 mg/ml protein; i.e., ~10% of the concentration in plasma. Since proteins in ASL are numerous and their concentrations are unknown, we used albumin as a generic polypeptide during microtitrations. While this is clearly an oversimplification, it is compatible with the early studies reporting synthesis and release of “albumin-like” protein from submucosal gland acinar cells (Jacquot et al., 1988). Albumin caused significant buffering at 1–10 mg/ml and would contribute to the buffer capacity of ASL, especially at low pH. Secreted mucins are the most abundant luminal glycoproteins and the main constituent of mucus (Rose et al., 1979), comprising 2–3% of the total mass of the mucus gel (20–30 mg/ml). Tethered mucins in brush-like structures may achieve similar concentrations in the periciliary layer (M. Rubinstein, pers. commun.). Microtitration of porcine gastric mucins at concentrations corresponding to the concentration of mucins in Calu-3 secretions suggest their buffering of Calu-3 fluid is negligible. Lysozyme was examined (Dubin et al., 2004; Joo et al., 2004) however its concentration was also too low little to provide significant buffer capacity, in agreement with a previous study (Kuramitsu and Hamaguchi, 1980). Finally, PO^=^_4_ was not detected in Calu-3 secretions using our methods, and microtitrations revealed that it would also contribute little to β at *in vivo* concentrations (Govindaraju et al., 1997). In summary, the present results suggest that HCO^−^_3_ and epithelially-derived proteins are the main buffers in Calu-3 secretions.

### Conflict of interest statement

The authors declare that the research was conducted in the absence of any commercial or financial relationships that could be construed as a potential conflict of interest.
