# Supplementary material for: The buffer capacity of airway epithelial secretions
Source: Front Physiol. 2014 Jun 3;5:188. doi: 10.3389/fphys.2014.00188 (PMC4042063; doi:10.3389/fphys.2014.00188)
Supplement: Supplementary file 1 [file DataSheet1.PDF]

SUPPLEMENTARY MATERIALS

METHODS: ANALYSES OF ELECTROLYTES AND ALBUMIN

Sodium and potassium ion concentrations were measured by indirect potentiometry utilizing glass ion-selective electrodes after dilution in high molar strength buffer. Chloride was measured using a solid state electrode. Total carbon dioxide and HCO_3^- were determined using a pH rate-of-change method which employs a glass carbon dioxide electrode in conjunction with a glass pH reference electrode. Total calcium concentration was determined by indirect potentiometry using a calcium ion selective electrode in a buffer that contains strong complexing agents to ensure a constant molar ratio between free and total calcium concentrations. Magnesium concentration was measured using a colorimetric timed endpoint method in which the sample was diluted in solution containing calmagite, and the change in absorbance at 520nm was measured when Mg and calmagite formed a stable chromogen. Total protein concentration was determined using the Bradford assay with BSA as a control. Bradford reagent (300 µl) was added to 10 µl of BSA solution or Calu-3 secretions in a cuvette, mixed for 30s, then incubated at 22 ºC for 30 min. Protein content was analyzed at 590nm using a spectrophotometer (Bio-Rad).
